# Correlation between surgical position and neck pain in patients undergoing thyroidectomy: a prospective observational study

**DOI:** 10.1186/s13741-024-00428-1

**Published:** 2024-07-15

**Authors:** Salvatore Pagliaro, Leonardo Rossi, Michela Meligeni, Letizia Catani, Riccardo Morganti, Gabriele Materazzi, Sohail Bakkar, Antonia Montanino, Danilo Pagliaro, Monica Scateni, Nicola Pagnucci

**Affiliations:** 1 University Hospital of Pisa, Pisa, Italy; 2 Medical, Molecular, and Critical Area Pathology, Department of Surgery, University of Pisa, Pisa, Italy; 3 The Hashemite University, Zarqa, Jordan; 4 Department of Translational Research and of New Surgical and Medical Technologies, University of Pisa, Pisa, Italy

**Keywords:** Neck hyperextension, Nursing care, Postoperative pain, Surgical position, Thyroid cancer, Thyroidectomy

## Abstract

**Background:**

Thyroid diseases are one of the most common health problems worldwide. Although they represent a necessary step in order to perform thyroidectomy, hyperextension of the neck can potentially increase postoperative pain. The aim of this study is to determine a correlation between the degree of neck hyperextension on the operative table and the postoperative pain in patients undergoing open thyroidectomy.

**Methods:**

Patients were prospectively enrolled from the cohort of patients operated at the Endocrine Surgery Unit of the University Hospital of Pisa, between May and July 2021. Both of patients who underwent total thyroidectomy or hemi-thyroidectomy were recruited. The following data were analysed in order to find a correlation with postoperative pain at 24 h: age, gender, type of surgery, BMI, operative time, and degree of neck extension.

**Results:**

Overall, 195 patients were enrolled. A direct, statistically significant correlation emerged between the degree of neck hyperextension and the postoperative pain 24 h after surgery, regardless of the pain of the surgical wound (*p* < 0.001; beta 0.270).

**Conclusions:**

A direct correlation emerges between neck tilt angle and postoperative neck pain. Moreover, total thyroidectomy (TT) predisposes more to postoperative neck pain, considering the type of surgery.

**Supplementary Information:**

The online version contains supplementary material available at 10.1186/s13741-024-00428-1.

## What is already known

•In a standard open thyroidectomy, patients are positioned with their necks hyperextended to facilitate exposure and visibility of the surgical field.

•Post-thyroidectomy hyperextension pain occurs in the posterior region of the neck and in shoulders area.

•One of the most important parameters for the patient to assess his satisfaction is postoperative pain

## What this paper adds

•Total thyroidectomy predisposes to postoperative neck pain.

•There is a statistically significant direct correlation between the degree of neck hyperextension and non-incisional postoperative neck pain 24 h.

## Introduction

Thyroid diseases are one of the most common health problems worldwide (Alyahya et al. 2021). Thyroid cancer (TC) is the most common malignant neoplasm of the endocrine system, and surgical treatment (total thyroidectomy or hemi-thyroidectomy, according to the standard guidelines) is the standard of care (Rodriguez-Torres et al. 2019). Overall, thyroidectomy is one of the most commonly performed surgical procedures, and, in experienced hands, the associated morbidity is relatively low (Lang and Lo 2010). In a standard open thyroidectomy, patients are positioned with their necks hyperextended to facilitate exposure and visibility of the surgical field (Serpell et al. 2003), as well as better access to the thyroid lodge (and consequently the noble structures contained therein, i.e. the RLN and parathyroid glands) (Serpell et al. 2003). Although it represents a necessary step, hyperextension of the neck can potentially increase postoperative pain (Serpell et al. 2003).

In literature, post-thyroidectomy neck pain is documented in more than 80% of cases (Rodriguez-Torres et al. 2019). Neck pain, stiffness, difficulty in neck and shoulder movement, and a feeling of suffocation or pressure are common symptoms in thyroidectomized patients (Alyahya et al. 2021; Rodriguez-Torres et al. 2019). Occasionally, a significant decrease in the range of movement of the neck, which may persist for more than 6 months after surgery, is documented (Rodriguez-Torres et al. 2019).

Post-thyroidectomy hyperextension pain occurs in the posterior region of the neck and in shoulders area; it reaches its higher peak in the first 24 h after surgery (Lang and Wong 2013; Shih et al. 2010). One of the most important parameters for the patient to assess his satisfaction is postoperative pain; moreover, it has got an important impact on postoperative recovery and analgesic consumption (with a direct influence on healthcare costs) (Lang and Wong 2013).

Overall, post-thyroidectomy pain is a widespread problem worldwide; however, few studies are currently dealing with the association between it and neck hyperextension. Furthermore, there is a lack of studies correlating an eventual reduction in neck hyperextension with a decrease in postoperative pain.

The aim of this study is to investigate the correlation between neck hyperextension and post-thyroidectomy pain.

## Methods

### Study design

A prospective observational cohort study has been realised in the period of May and July 2021.

### Participants

Patients were prospectively included from the cohort of patients operated on at the Endocrine Surgery Unit of the University Hospital (hidden detail), between May and July 2021.

#### Inclusion criteria

These are patients undergoing total thyroidectomy (TT) or hemi-thyroidectomy (HT) and adult patients aged over 18 years.

#### Exclusion criteria

These are patients with proven diseases of the cervical spine (e.g. cervicalgia, cervical disc herniation, spondyloarthrosis), patients undergoing operations other than total or partial thyroidectomy, patients undergoing laterocervical or central compartment lymph node dissection, patients undergoing robot-assisted transaxillary thyroidectomy, and patients undergoing minimally invasive video-assisted thyroidectomy (MIVAT).

The following data were scrutinised in order to find a correlation with postoperative pain at 24 h: age, gender, type of surgery, BMI, operative time, and degree of neck extension.

### Methods of data collection

Data collection took place in three times.

#### Time 0 (T0): before surgery

Collection of informed consent and demographic and clinical data (age, sex, BMI, type of surgery — total thyroidectomy and partial thyroidectomy) and neck length measurement (measured in centimetres from the tip of the mastoid process up to the ipsilateral sternoclavicular joint).

#### Time 1 (T1): during surgery

**Fig. 1:**
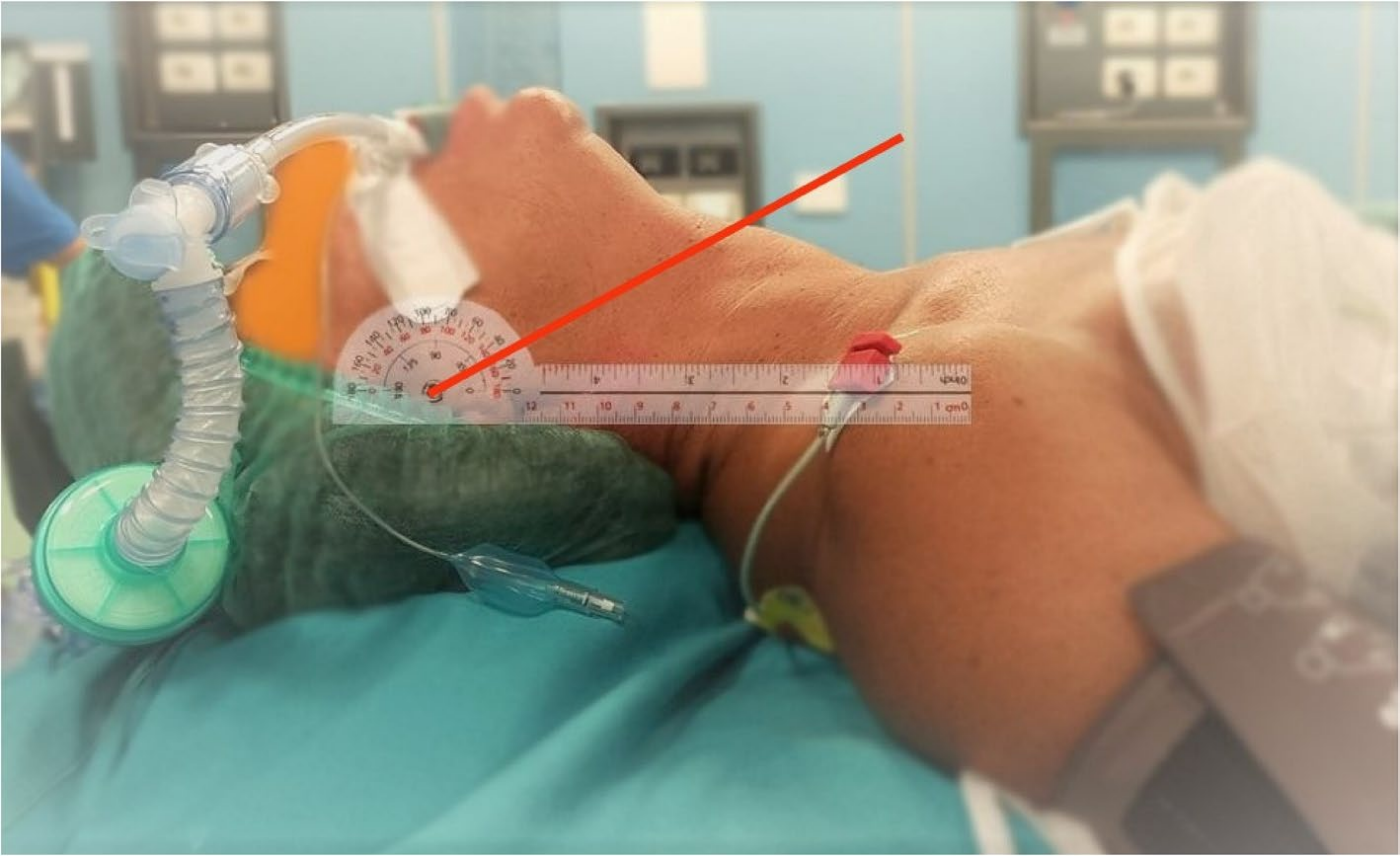
Measurement of the neck extension angle using the orthopedic goniometer

The patient, sedated and intubated, was placed in supine position with the neck hyperextended with the arms along the body. For positioning, a shoulder roll was not necessary because the operating tables allowed for the headboard to be tilted. A 4-cm head doughnut cushion has been positioned (as customarily) to stabilise the neck and head during the procedure and prevent unintended movements. A researcher was in charge of measuring the extension angle of the neck using an orthopaedic goniometer (absolute error maximum 1°), with a circular scale (with measurements in degrees from 0 to 360 clockwise and anticlockwise), characterised by two arms (each 30 cm long) and a circular graduated part (both increasing and decreasing). Of the two arms, one acted as the “fixed arm”, i.e. the 0 point, while the other was rotated until the angle of neck extension was reached. For the measurement, it was necessary to consider four fixed landmarks to draw imaginary axes on which the arms of the instrument could lay. The “fixed arm” of the orthopaedic goniometer was placed on the inter-articular line of the body segment and kept stationary (i.e. the imaginary axis of conjunction between the acromion and the patient’s mandibular-temporal joint). We rephrased the mobile arm measurement for better clarity: The “movable arm” of the goniometer was rotated until it aligned with the imaginary axis connecting the apex of the sternocleidomastoid muscle to its insertion points — specifically, between the sternal head, the mastoid process of the temporal bone, and the lateral third of the superior nuchal line (see Fig. 1).

The following data were extracted at this stage: the neck hyperextension angle and the surgery duration.

#### Time 2 (T2): after surgery

At 24 h after surgery, the presence of non-incisional postoperative neck pain in the cervical spine was detected using the Numerical Rating Scale (NRS), scale comprising values from 0 (“absolutely no pain”) to 10 (“worst pain imaginable”).

### Ethical aspects

The present study was approved by the ethics committee of the University Hospital of Pisa (Approval number 731.2021); Participant anonymity was ensured by replacing their names with an alphanumeric code. Patients who met the inclusion criteria were free to decide whether to take part in the research after receiving all the necessary oral and written information to understand the aim of the study and the respective risks and benefits. The information sheet clearly stated that refusal to take part in the study would not affect in any way the quality of the care provided nor the good relations with the health professionals, ensuring the confidentiality of this information. A written consent was obtained from each participant.

### Statistical analysis

Categorical data were described by absolute and relative (%) frequency and continuous data by mean and standard deviation. To evaluate the factors influencing “postoperative pain”, a model based on the multiple linear regression was performed. Regression coefficient with 95% CI and “partial correlation coefficient” (beta) were also indicated. Significance was fixed at 0.05, and all analyses were carried out with IBM SPSS Statistics for Macintosh, Version 28.0, Armonk, NY, USA.

## Results

Overall, 211 patients were assessed for eligibility. Sixteen patients were excluded from the study according to the exclusion criteria abovementioned: 8 presented cervical spine disease, 2 underwent parathyroid surgery, and 6 underwent MIVAT. A total of 195 patients were enrolled. The mean age of the study participants was 48 years (*SD* 15), and the prevalent sex was female (*n* = 139; 71.3%). The mean postoperative pain was 3.1 (*SD* 2.5) on NRS. Total thyroidectomy was the prevalent type of surgery with 154 interventions (59%). TT had an average duration of 58 (*SD* 11.6) minutes, while HT had an average duration of 31 min (*SD* 8.3). Characteristics of enrolled patients are summarised in Table 1.

**Table 1:** Characteristic of enrolled patients

	Mean (SD)
Age (y)	48 (15)
BMI	25.9 (4.9)
Postoperative pain (NRS)	3.1 (2.5)
Surgery duration (min)	52 (18)
Inclination angle (degrees)	18.9 (6)
Neck length (cm)	10.4 (1.4)
	*N* (%)
Gender	
*Female*	139 (71.3)
*Male*	56 (28.7)
Type of sugery	
*HT*	41 (21)
*TT*	154 (79)

*H* hemithyroidectomy, *TT* total thyroidectomy, *BMI* body mass index

After surgery, all patients were monitored in the recovery room.

A direct correlation emerges between neck tilt angle and postoperative neck pain (*p* < 0.001; beta 0.270). Taking into consideration the type of surgery, total thyroidectomy (TT) predisposes to postoperative neck pain (*p* 0.052; beta 0.137). No other correlations have been found (Table 2).

**Table 2:** Multivariate analysis (multiple linear regression) of the factors influencing postoperative pain

Factor	RC	95% *CI*	Beta	*p*-value
Age	0.013	− 0.01; − 0.04	0.075	0.295
Gender: (0) M, (1) F	− 0.157	− 0.94; 0.63	− 0.028	0.694
BMI	0.035	− 0.04; 0.11	0.069	0.341
Surgery duration	− 0.002	− 0.02; 0.02	− 0.011	0.875
Inclination angle	0.112	0.05; 0.17	0.270	< 0.001
Neck length	− 0.162	− 0.42; 0.09	− 0.090	0.211
Type of surgery: (0) HT, (1) TT	0.838	− 0.01; 1.68	0.137	*0.052*
(Constant)	0.619	− 3.3; 4.5		0.753

*RC* regression coefficient, beta, partial correlation coefficient, *HT* hemithyroidectomy, *TT* total thyroidectomy, *M* male, *F* female, *BMI* body mass index

## Discussion

The prevention and treatment of postoperative pain are a long-debated issue which is attracting more and more attention. Thyroidectomy is associated both to postoperative pain located in the surgical wound and in the posterior neck. In particular, in 2015, Han et al. (Han et al. 2006) reported that 80% of patients who underwent thyroid surgery complain about posterior neck pain postoperatively. Posterior neck pain is probably due to the position of the patient on the operative bed with the neck hyperextended (Han et al. 2006). Indeed, the operating table is equipped with a manually adjustable headrest to calibrate neck extension keeping the head in a stable position.

Postoperative neck pain, a distinct entity from surgical pain located at the wound, occurs commonly after thyroidectomy and is treated with analgesics. As wound pain occurs immediately after surgery, it usually lasts for about 48 h (Genc et al. 2019).

In our study, the inclination angle of hyperextension on the operating table was significantly associated with postoperative neck pain (*p* < 0.001; beta 0.270), indicating a direct correlation. Our results are consistent with the study by Lang et al. (Lang et al. 2015), who compared postoperative pain in patients scheduled for thyroidectomy with or without neck hyperextension on the operating table. The authors reported that postoperative pain was associated with neck hyperextension, although they did not correlate it with the degree of inclination.

The correlation between the angle of inclination and postoperative neck pain can be attributed to several factors. Intraoperative positioning, which involves hyperextension of the neck, can lead to unnatural postures and tension in the neck muscles, trapezius, and brachial plexus. The duration of the surgery and the use of anaesthetics combined with muscle relaxants contribute to the relaxation and subsequent tension of the neck and shoulder muscles, as well as the nerves of the brachial plexus. Surgical manoeuvres to improve the visibility of the operative field and access to the thyroid lobes, as well as to ensure adequate exposure of vital structures like the laryngeal nerve and parathyroids, can also contribute to discomfort (Lang et al. 2015). Additionally, the use of retractors and the pressure and tension applied to the skin, subcutaneous tissues, and muscle fascia, along with manipulation of anatomical structures around the trachea and the inherent elasticity of the neck, play a role.

Although no other parameter in our analysis was correlated with postoperative pain, a trend toward a correlation with the type of surgery was observed: TT was associated with the onset of postoperative neck pain, though it did not achieve statistical significance (*p* = 0.052). TT predisposes patients to postoperative neck pain. The duration of this surgery necessitates maintaining the neck in a hyperextended position on the operating table for a longer period compared to HT, maintaining cervical muscles and ligaments stretched.

The absence of statistically significant correlations, aside from the one between the angle of inclination and postoperative neck pain, underscores the critical importance of this factor in developing this adverse outcome.

Furthermore, our findings suggest that as the angle of neck inclination increases, postoperative pain will likely increase. We hope that in the future, larger studies will explore more in depth this aspect.

Unfortunately, our study fails to establish an ideal range of neck inclination to avoid postoperative neck pain; however, it could be recommended not to overextend the neck of patients undergoing open thyroidectomy. Indeed, overextension does not significantly improve the access to the thyroid gland, as reported by Serpell et al. (Serpell et al. 2003); on the other hand, it may promote the onset of postoperative pain and discomfort (Lang et al. 2015) which may persist for several days and may negatively affect daily life (Rodriguez-Torres et al. 2019; Lang and Lo 2010; Serpell et al. 2003; Lang and Wong 2013; Shih et al. 2010; Han et al. 2006; Genc et al. 2019; Lang et al. 2015).

The management of postoperative pain is a serious issue. It is of primary importance to implement a bundle of pain prevention that focuses on the early identification of patients at risk (i.e. patients reporting neck stiffness, not engaging in sports, experiencing sporadic episodes of stiff neck without documented pathologies) to be addressed to perform head-neck stretching exercises to increase the degree of elasticity of neck muscles (Takamura et al. 2005). These exercises may lead to a decrease in the request for postoperative analgesics, limiting the unnecessary use of opioid drugs (Ferrell et al. 2021). Moreover, pain management improves postoperative comfort and patient satisfaction; it aids recovery and promotes rapid discharge.

Strategies reported in literature to treat postoperative neck pain include bilateral great occipital nerve blockade with bupivacaine (Han et al. 2006), intraoperative transcutaneous electrical nerve stimulation applied on the trapezius muscle (Park et al. 2015), and physiokinesiotherapy (Genc et al. 2019). Anyway, a further effort to assess factors associated with neck pain should be made in order to prevent it rather than treating it.

This study’s strengths include using objective measurement tools, such as the orthopaedic goniometer, a detailed data collection process, and analysis, which enhance the reliability of the results. A variety of validated tools are available for detecting pain, but most of the literature focuses on the NRS scale, considering it as the most popular. Similarly, this scale has been used to measure pain in previous studies in the field of thyroidectomy (Jo et al. 2021; Shrestha et al. 1976; Thorsen et al. 2022). Additionally, the study was conducted at a European reference centre for thyroidectomies, allowing for precise measurement of the inclination angle of hyperextension on the operating table. Other positive aspects include the uniformity of operating times, the sample’s consistency, and the healthcare workers’ standardised work methods.

## Conclusion

The present study demonstrated a statistically significant direct correlation (*p* < 0.001; beta 0.270) between the degree of neck tilt and non-incisional postoperative neck pain 24 h after surgery in patients undergoing open thyroidectomy. Moreover, a trend correlation also emerges regarding to the type of surgery that should not be underestimated (*p* 0.052; beta 0.137), with total thyroidectomy associated with postoperative neck pain.

Anyway, this study harbours limitations. Indeed, an ideal range of neck hyperextension has not been identified. Further prospective future studies may clarify this important and debated topic, and a protocol for pain prevention and selection of patients at risk may hopefully be established.

### Supplementary Information

Supplementary Material 1.

## Data Availability

The datasets used and/or analysed during the current study are available from the corresponding author on reasonable request.
